# Balance Performance After Mild Traumatic Brain Injury in Children and Adolescents: Instrumented BESS in the Acute Situation and Over Time

**DOI:** 10.3390/jcm14051666

**Published:** 2025-02-28

**Authors:** Nils K. T. Schönberg, Johanna Wagner, Korbinian Heinrich, Ida Kandler, Tobias Graf, Rieke Böddeker, Lea Zinke, Nicole Fabri, Julia Wilke, Florian Hoffmann, A. Sebastian Schröder, Anne-Sophie Holler, Alexandra Fröba-Pohl, Oliver Muensterer, Doreen Huppert, Matthias Hösl, Florian Heinen, Michaela V. Bonfert

**Affiliations:** 1 Department of Pediatric Neurology and Developmental Medicine and LMU Center for Children with Medical Complexity, LMU Munich, Dr. von Hauner Children’s Hospital, 80336 Munich, Germany; n.schoenberg@campus.lmu.de (N.K.T.S.); michaela.bonfert@med.lmu.de (M.V.B.); 2 Pediatric Intensive Care and Emergency Medicine, Dr. von Hauner Children’s Hospital, 80336 Munich, Germany; 3 Center for Child Neurology and Social Pediatrics, Child Centre Maulbronn, 75433 Maulbronn, Germany; 4 Department of Pediatric Surgery, Dr. von Hauner Children’s Hospital, Ludwig-Maximilians-University (LMU), 80336 Munich, Germany; 5 German Center for Vertigo and Balance Disorders, Ludwig-Maximilians-University (LMU), LMU Hospital, 80539 Munich, Germany; 6 Gait and Motion Analysis Laboratory, Schön Klinik Vogtareuth, 83569 Vogtareuth, Germany; 7 Institute for Transition, Rehabilitation and Palliation, Paracelsus Medical University Salzburg, 5020 Salzburg, Austria

**Keywords:** traumatic brain injury, concussion, persistent post-concussive symptoms, post-concussion syndrome, postural control

## Abstract

**Background**: Mild traumatic brain injury (mTBI) in the pediatric population is a significant public health concern, often associated with persistent post-concussion symptoms, including postural instability. Current tools for assessing postural control, such as the Balance Error Scoring System (BESS), lack integration with objective metrics. Incorporating force plate sensors into BESS assessments may enhance diagnostic accuracy and support return-to-play or sports decisions. This study evaluates postural performance in children with mTBI compared to controls using an instrumented BESS and examines recovery trajectories after mTBI. **Methods**: This prospective, longitudinal study included 31 children with mTBI (12.01 ± 3.28 years, 20 females) and 31 controls (12.31 ± 3.27 years, 18 females). Postural control was assessed using an instrumented BESS protocol during standing on a ground reaction force plate at three timepoints: within 72 h post injury (T1), at two weeks (T2), and three months after trauma (T3). Posturographic parameters derived from the displacement of the center of pressure included the ellipse area, path length, and mean velocity in the anterior–posterior and medio–lateral directions. Symptom burden was monitored using the Post-Concussion Symptom Inventory (PCSI). **Results**: The BESS total scores did not differ significantly between the groups at any timepoint. A significant reduction in BESS errors over time was observed exclusively in the two-legged stance on a soft surface (*p* = 0.047). The instrumented BESS revealed higher body swaying in the mTBI group compared to controls, particularly under demanding conditions. Significant between-group differences were most frequently observed in single-leg soft surface (38% of comparisons) and two-legged soft surface stances (29%). In those cases, path length and mean velocity differed between groups, respectively. Ellipse area did not show significant differences across conditions. **Conclusions**: An instrumented BESS has the potential to enhance the detection of subtle postural deficits in pediatric mTBI patients. Specifically, more demanding conditions with altered sensory-proprioceptive input and path length as an outcome measure should be focused on. This study underscores the need for tailored and age-appropriate objective and quantitative balance assessments to improve diagnostic precision in pediatric mTBI populations.

## 1. Introduction

Traumatic brain injury (TBI) in the pediatric population is a significant global public health concern, with incidences exceeding three million cases annually [1]. Most of these cases are classified as mild TBI (mTBI), characterized by the presence of at least one physical-neurological, cognitive, or emotional symptom without a lasting impairment of consciousness, and consequently a Glasgow Coma Scale (GCS) Score of >12 at the time of presentation [1,2,3,4]. Typically, symptoms resolve within a few days, but a protracted trajectory is expected in 5 to 30% of patients who experience persistent post-concussive symptoms [4,5,6]. In this context, dizziness and balance disturbances are common post-mTBI symptoms and may cause an instability in posture during standing and moving, increasing the risk of tripping or falling, resulting in secondary injuries during daily activities like play and sports [7,8,9,10,11]. This elevated risk might result in a timely second mTBI, with a higher likelihood of more prolonged recovery [12,13,14]. Furthermore, postural and vestibular symptoms have previously been reported as risk factors for persistent post-concussive symptoms (PPCS) and may play an important role in identifying vulnerable populations [15,16]. Consequently, postural instability significantly informs recommendations for a more conservative return to physical activity and sports after mTBI [17,18].

Impaired postural control following mTBI arises from complex disturbances in central integrative mechanisms involving the visual, proprioceptive, vestibular, and sensory-motor systems [19,20]. Currently, there is no reliable instrument available that comprehensively assesses the multifaceted symptoms and pathophysiology in balance issues of pediatric patients after mTBI [12,21,22]. While diagnostic tools like the Balance Error Scoring System (BESS), endorsed by the Concussion in Sport Group (CISG) and the National Athletic Trainers’ Association (NATA), are widely used in sports medicine to support return-to-play decisions post-head injury, their use in the pediatric point-of-care setting has not been established yet [18,23,24]. Instrumented postural control assessment using force plates is another recognized strategy in adult neurology and sports medicine to objectively assess postural performance [25,26,27,28,29]. Integrating the BESS with a ground-reaction force plate to measure body swaying (instrumented BESS) may provide a more comprehensive assessment of postural stability in pediatric mTBI patients [30,31]. While postural control relies on a complex interplay of sensory systems and central integrative mechanisms, body sway measures have been widely recognized as robust indicators of postural performance. To ensure an easily interpretable and clinically applicable assessment tool, particularly in point-of-care settings, we prioritized body sway metrics in this investigation. This approach aims to support clinicians in making informed decisions regarding postural impairment in mTBI patients [32,33].

To establish robust diagnostic measures for instrumented BESS protocols, particularly in the younger pediatric populations, clinical posturographic data are necessary. This pilot study aimed to investigate the postural performance of pediatric patients after mTBI (patient group; PG) at three different timepoints after their injury and compare their postural performance to data acquired in a control group of peers (CG).

The hypothesis of the presented work was that postural performance of the PG would be poorer compared to that of the CG (as indicated by higher levels of body swaying) due to the alteration of processing visual, proprioceptive, and vestibular information following mTBI [19,21,34]. Additionally, we anticipated a reduction in body swaying within the PG from T1 to T2 to T3, reflecting a recovery of postural control after trauma [35,36].

## 2. Methods

This exploratory, prospective, non-interventional, longitudinal cohort study was approved by the local institutional review board (vote numbers 21-0448 and 20-160). Written informed consent was obtained from all study participants and their legal guardians prior to study inclusion. This study was conducted in accordance with the ethical principles established in the Declaration of Helsinki (World Medical Association).

### 2.1. Study Population

Participants were recruited from the emergency department, surgical ward, or intensive care unit of a tertiary pediatric hospital in a metropolitan area. Eligible participants were individuals aged 6 to 17 years who had been diagnosed with a first-time mTBI within 72 h after trauma by a qualified medical practitioner according to the definition of mTBI proposed by Lefevre et al. in 2021 [2]. The exclusion criteria encompassed prior moderate or severe TBI at any timepoint, an mTBI within the last 24 months, trauma resulting from assault, prematurity (<34 weeks of pregnancy), pre-existing physician diagnosed neurological or psychiatric conditions (e.g., epilepsy, cerebral palsy, depression), and concomitant injuries that rendered balance testing unfeasible (e.g., lower limb fractures, postural impairment due to the weight of a hard cast on an upper limb fracture).

The control group comprised children who were treated for mTBI at our tertiary pediatric center over the past 10 years and who initially did not exhibit any disturbances in postural control. Eligibility criteria included children who had sustained mTBI with complete, non-protracted clinical recovery—assessed using the Kings Outcome Scale for Childhood Head Injury (KOSCHI)—and who were between 6 and 17 years of age at the time of recruitment [37]. The average time since mTBI was 62.33 ± 35.61 months. Given the high prevalence of mTBI in the pediatric population, including many unreported cases, a history of mTBI is expected in a significant proportion of pediatric cohorts [38,39]. Therefore, our control group served as a representative peer cohort. To ensure that the control group included only asymptomatic individuals without residual symptoms or a history of postural impairments, a stringent screening process was conducted, including a comprehensive assessment by a pediatric neurologist.

### 2.2. Assessment

**Figure 1:**
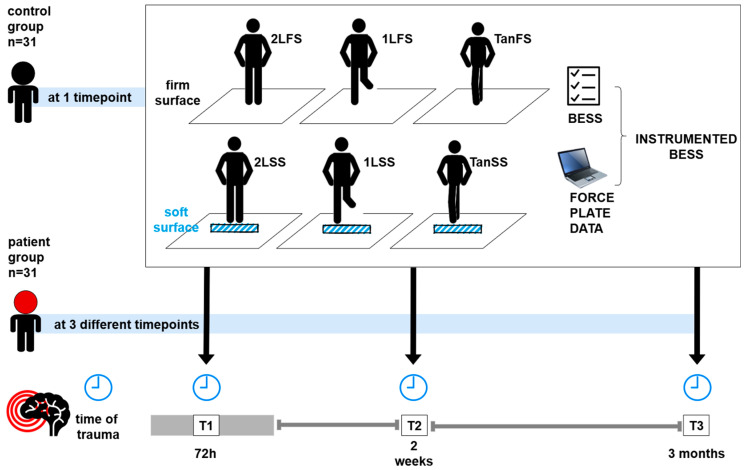
Study design (figure created using diagrams.net).

Study inclusion took place within the initial 72 h following the traumatic incident, and the participants underwent assessments of their postural control at three timepoints: within 72 h (on average: 1.27 ± 0.55 days) (T1), at 2 weeks (18.62 ± 7.07 days) (T2), and 3 months (110.80 ± 13.70 days) after the trauma (T3) (Figure 1, Table 1).

The BESS testing protocol was carried out by each participant while standing quiet on a force plate (Leonardo Mechanograph^®^GRFP LT force plate, Novotec Medical GmbH, Pforzheim, Germany) across all assessments (plate measurements: 660 mm × 660 mm × 70 mm). The PG was tested three times across the study’s timepoints (T1–T3), while the CG was assessed once in a cross-sectional approach. The participants received age-appropriate instructions for the posturographic examination in each testing position. In the event of discomfort, participants could stop the exam at any given moment. Six distinct testing conditions, each lasting 20 s, were tested for every participant while standing barefoot on the force plate, with their hands resting on their iliac crests. Herein, three standing tasks were measured once with and without a foam surface on the ground, according to the BESS protocol. Prior to testing, the dominant foot was determined via a ball kicking exercise. The test conditions comprised:

(1) Firm surface, two-legged stance–feet hip-width apart **(2LFS)**;
(2) Firm surface, one-legged stance on the non-dominant foot **(1LFS)**;
(3) Firm surface, tandem stance with non-dominant foot in back **(TanFS)**;
(4) Soft surface, two-legged stance **(2LSS)**;
(5) Soft surface, one-legged stance **(1LSS)**;
(6) Soft surface, tandem stance with non-dominant foot in back **(TanSS)**.

For soft surface testing, a foam pad (480 mm × 400 mm × 60 mm, density: 38.6 kg/m^3^, AIREX^®^, Sins, Switzerland) was placed on the force plate. Two examiners (physician and research assistant) trained in BESS testing documented each BESS mistake. BESS mistakes were defined as moving the hands off the iliac crests, opening the eyes, every step stumble or fall, abduction or flexion of the hip beyond 30°, lifting the forefoot or heel off the testing surface, and remaining out of the proper testing position for more than 5 s [40]. BESS errors were recorded while simultaneously collecting posturographic data on behalf of the force plate. Posturographic data were collected at a sampling rate of 500 Hz. The raw data were subsequently filtered using a low-pass filter with a cutoff frequency of 100 Hz to minimize high-frequency noise while preserving the clinically relevant frequency content of the CoP signal. Previous research has demonstrated that the majority of postural control dynamics, including high-frequency adjustments, occur at frequencies below 10 Hz, with minimal contributions from frequencies above 20 Hz [41,42,43]. A low-pass cutoff of 100 Hz was therefore sufficient to capture these movements without loss of meaningful information. This approach aligned with established signal processing guidelines for posturographic studies and ensured robust analysis of CoP dynamics [44,45]. Leonardo Mechanography v4.4 software was utilized to analyze each dataset (BAS Edition V4.4b03.42, Novotec Medical GmbH, Pforzheim, Germany). The participant’s center of pressure (CoP), the point of application of the ground reaction force onto the force plate, was focused on as primary measure. The trajectory of the CoP is frequently used to evaluate postural control, because it indirectly represents the participant’s capacity to maintain the body’s center of mass over the base of support. The parameters derived from the CoP measurements comprised the ellipse area (EA; cm^2^) (accounting for 90% of COP points during body excursion), using the best fitting ellipse to gain maximum coverage as a measure of sway magnitude; path length (PL; mm), including the path length in the anterior–posterior direction (PL AP; mm) and the path length in the medio–lateral direction (PL ML; mm) as indicators of sway trajectory; and mean velocity (MV; mm/s) in the anterior–posterior direction (MV AP; mm/s) and in the medio–lateral direction (MV ML; mm/s) as indicators of body sway responsiveness.

To assess the burden of symptoms after mTBI within the PG, we employed the validated Post-Concussion Symptom Inventory (PCSI) authored by Gioia, Janusz, Vaughan & Sady in its German translation by von Steinbuechel and colleagues for participants in their respective age groups (PCSI for children aged 5–8 years, 8–12 years, and 12–18 years) at T1, T2, and T3 [46,47,48,49].

### 2.3. Data Management and Statistical Analysis:

The clinical data were documented within paper-based case report forms/questionnaires and were centrally entered into an electronic database (Microsoft Excel for Windows Version 2024, Microsoft Corp., Redmond, WA, USA). Participants in the PG who were lost to follow-up were excluded from the statistical analysis. There were no missing data in either the PG or CG. Statistical analyses were conducted utilizing the Statistical Package for the Social Sciences (IBM SPSS Statistics for Windows Version 29.0.2.0, IBM Corp., Armonk, NY, USA). The level of significance was set to *p < 0.05.*

The Shapiro–Wilk test was employed to verify the statistical distribution of all obtained datasets. Between-group differences between the PG and the CG for both the BESS and instrumented BESS were determined utilizing t-tests for parametric data and Mann–Whitney U-tests for non-parametric data. Comparative within-group analysis of the BESS and instrumented BESS at T1, T2, and T3 within the PG was carried out utilizing repeated measures ANOVA testing. Significant ANOVA results were further analyzed utilizing Tukey’s range post hoc testing. Comparative within-group analysis of PCSI in their age-groups across timepoints was conducted utilizing Friedman testing with post hoc pairwise comparisons via the Wilcoxon signed-rank test with Bonferroni correction. No quantitative statistical analysis was conducted in the 5–7 years age group due its small sample size (n = 3).

## 3. Results

### 3.1. Cohort Characteristics

Thirty-one patients (20 females) aged 12.01 ± 3.28 (range 6–17) years and thirty-one (18 females) controls aged 12.31 ± 3.27 (range 6–17) years took part in this study (Table 1). The burden of symptoms after mTBI in the PG was measured on behalf of the PCSI questionnaire and declined over time in all groups (Supplemental Table S8). Patients between:

- 5 to 7 years (*n* = 3) exhibited mean PCSI-rapid scores of: **T1** = 4.7 ± 0.5, **T2** = 1.0 ± 0.0, and **T3** = −2.7 ± 5.9 (no statistical analysis feasible due to low sample size)
- 8 to 12 years (*n* = 14) exhibited mean PCSI-rapid scores of: **T1** = 9.1 ± 7.4, **T2** = 1.1 ± 4.4, and **T3** = 1.4 ± 4.1 (Friedman test χ2 = 16.34, *p* < 0.001)
- 13 to 17 years (*n* = 14) exhibited mean PCSI-rapid scores of: **T1** = 18.3 ± 15.2, **T2** = 9.9 ± 13.5, and **T3** = 5.4 ± 10.9 (Friedman test χ2 = 6.29, *p* = 0.043)

**Table 1:** Cohort characteristics.

Patient Group (PG)			
**Sex**		**Age in Years**	
male	female	mean (sd)	median (iqr)
11	20	12.01 (3.28)	12.06 (5.08)
	**Time after mTBI at participation**		
T1	1.27 ± 0.55 days after mTBI		
T2	18.62 ± 7.07 days after mTBI		
T3	110.80 ± 13.70 days after mTBI		
*n*	**5 to 7 Years**	**8 to 12 Years**	**13 to 17 Years**
	3	14	14
PG T1 PCSI-RAPID	4.7 ± 0.5	9.1 ± 7.4	18.3 ± 15.2
PG T2 PCSI-RAPID	1.0 ± 0.0	1.1 ± 4.4	9.9 ± 13.5
PG T3 PCSI-RAPID	−2.7 ± 5.9	1.4 ± 4.1	5.4 ± 10.9
**Control Group**			
**Sex**		**Age in Years**	
male	female	mean (sd)	median (iqr)
13	18	12.31 (3.27)	14 (4.5)

### 3.2. BESS

The highest total BESS score in the PG was recorded at T1 (22.55 ± 11.88), which slightly numerically decreased at T2 (19.45 ± 9.84) and remaining at this level at T3 (19.10 ± 8.45), ANOVA: F = 1.115, *p* = 0.332 (Table 2 and Supplemental Table S7). The total BESS score in the CG was 21.29 ± 5.78, which did not significantly differ from that of the PG at T1 (*p* = 0.604), T2 (*p* = 0.109), or T3 (*p* = 0.210). The fewest BESS errors were reported during 2LFS (PG T1: 0.35 ± 1.77, T2: 0.32 ± 0.18, T3: 0.07 ± 0.25; CG: 0); most BESS errors occurred during 1LSS for both groups (PG T1: 7.84 ± 2.53, T2: 8.19 ± 1.89, T3: 8.03 ± 2.11; CG: 8.61 ± 1.50) (Table 2). In the PG, the BESS scores significantly decreased under the 2LSS condition across the three timepoints (F = 3.171, *p* = 0.047) from T1 (1.35 ± 2.55) to T2 (0.55 ± 1.81; *p* = 0.041) as well as from T2 to T3 (0.20 ± 0.54; *p* = 0.031) (Supplemental Table S7). Under the 2LSS condition, the PG demonstrated fewer errors than the CG at T2 (*p* < 0.001) and at T3 (*p* < 0.001) (Supplemental Table S7).

### 3.3. Instrumented BESS

**Figure 2:**
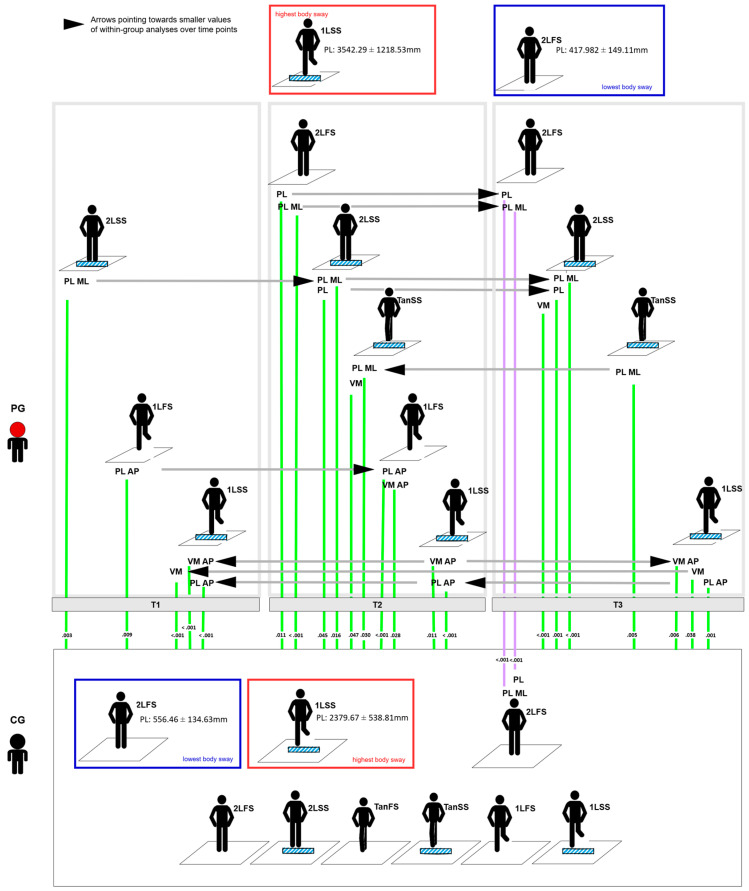
Significant differences between mTBI patients and controls in the posturographic assessment across the three study visits. The analysis of all of the 7 outcome measures under all of the 6 testing conditions resulted in *n* = 24 significant findings. Green lines mark measures that were higher in the PG than in the CG; the violet color marks measures that were lower in the PG than in the CG. Positions with lowest (blue) and highest (red) body swaying in each group are marked. For detailed data, please refer to Table 3 (figure created using diagrams.net).

The highest body swaying for the PG was observed in the 1LSS position at T2 (PL: 3542.29 ± 1218.53 mm). Similarly, in the CG, the highest body swaying was documented in the 1LSS position (PL: 2379.67 ± 538.81 mm). In the PG, the lowest body swaying was recorded at T3 in the 2LFS position (PL: 417.982 ± 149.11 mm). Again, in the CG, the lowest body swaying was also observed in the 2LFS position (PL: 556.46 ± 134.63 mm) (Table 3, Figure 2).

#### 3.3.1. Two-Legged Stance on Firm Surface (2LFS)

No significant within-group differences were identified for any of the variables in the PG across the three timepoints. Significant between-group differences were identified for several variables when comparing the PG at T1, T2, and T3 with the CG: **PL** at T2 (*p* = 0.011, higher in the PG) and T3 (*p* < 0.001, higher in the CG). **PL ML** at T2 (*p* < 0.001, higher in the PG) and T3 (*p* < 0.001, higher in the CG) (Table 4, Figure 2, Supplemental Table S1).

#### 3.3.2. Two-Legged Stance on Soft Surface (2LSS)

Significant within-group differences were observed in the PG across the three timepoints for **PL ML** (ANOVA: F = 4.421, *p* = 0.015), driven by an increase from T1 to T3 (*p* = 0.030). Significant between-group differences were observed for **PL** at T2 (*p* = 0.045) and T3 (*p* = 0.001), with the PG demonstrating higher PL than the CG. **PL ML** was significantly higher in the PG than in the CG at all three timepoints (T1: *p* = 0.003, T2: *p* = 0.016; T3: *p* < 0.001). Additionally, **VM** was significantly higher in the PG at T3 (*p* <.001) (Table 4, Figure 2, Supplemental Table S2).

#### 3.3.3. Tandem Stance on Firm Surface (TanFS)

No significant within-group or between-group differences were identified for any of the variables in the PG across timepoints (Table 4, Figure 2, Supplemental Table S3).

#### 3.3.4. Tandem Stance on Soft Surface (TanSS)

Significant within-group differences were observed in the PG across timepoints for **PL ML** (ANOVA: F = 4.150, *p* = 0.020), driven by an increase from T1 to T2 (*p* = 0.048) and T3 (*p* = 0.030), respectively. Significant between-group differences were observed for **PL ML** at T2 (*p* = 0.030) and T3 (*p* = 0.005), with the PG demonstrating higher PL ML than the CG. In addition, **VM** was significantly higher in the PG at T2 (*p* = 0.047) (Table 4, Figure 2, Supplemental Table S4).

#### 3.3.5. One-Legged Stance on Firm Surface (1LFS)

No significant within-group differences were identified in the PG across timepoints. Significant between-group differences were observed for **PL AP** at T1 (*p* = 0.009) and T2 (*p* < 0.001), with the PG demonstrating higher PL AP. In addition, **VM AP** was significantly higher in the PG at T2 (*p* = 0.028) (Table 4, Figure 2, Supplemental Table S5).

#### 3.3.6. One-Legged Stance on Soft Surface (1LSS)

No significant within-group differences were identified in the PG across timepoints. Significant between-group differences were observed for **PL AP** at all timepoints (*p* < 0.001), with the PG demonstrating higher PL AP. Additionally, **VM** was significantly higher in the PG at T1 (*p* < 0.001) and T3 (*p* = 0.038). Further, **VM AP** was significantly higher in the PG at all three timepoints (T1: *p* < 0.001; T2: *p* = 0.011; T3: *p* = 0.006) (Table 4, Figure 2, Supplemental Table S6).

**Table 4:** (**a**) Significant in-between group differences (between patient and control group) using the instrumented BESS. (**b**) Prevalent between-group significances.

(a)					
Condition	Variable		Timepoint	*p*-Value	Direction
two-legged stance on firm surface (2LFS)	Path Length		T2	0.011	Higher in PG
two-legged stance on firm surface (2LFS)	Path Length		T3	<0.001	Higher in CG
two-legged stance on firm surface (2LFS)	Path Length ML		T2	<0.001	Higher in PG
two-legged stance on firm surface (2LFS)	Path Length ML		T3	<0.001	Higher in CG
two-legged stance on soft surface (2LSS)	Path Length		T2	0.045	Higher in PG
two-legged stance on soft surface (2LSS)	Path Length		T3	0.001	Higher in PG
two-legged stance on soft surface (2LSS)	Path Length ML		T1	0.003	Higher in PG
two-legged stance on soft surface (2LSS)	Path Length ML		T2	0.016	Higher in PG
two-legged stance on soft surface (2LSS)	Path Length ML		T3	<0.001	Higher in PG
two-legged stance on soft surface (2LSS)	Velocity (mean)		T3	<0.001	Higher in PG
tandem stance on soft surface (TanSS)	Path Length ML		T2	0.030	Higher in PG
tandem stance on soft surface (TanSS)	Path Length ML		T3	0.005	Higher in PG
tandem stance on soft surface (TanSS)	Velocity (mean)		T2	0.047	Higher in PG
one-legged stance on firm surface (1LFS)	Path Length AP		T1	0.009	Higher in PG
one-legged stance on firm surface (1LFS)	Path Length AP		T2	<0.001	Higher in PG
one-legged stance on firm surface (1LFS)	Velocity (mean) AP		T2	0.028	Higher in PG
one-legged stance on soft surface (1LSS)	Path Length AP		T1	<0.001	Higher in PG
one-legged stance on soft surface (1LSS)	Path Length AP		T2	<0.001	Higher in PG
one-legged stance on soft surface (1LSS)	Path Length AP		T3	0.001	Higher in PG
one-legged stance on soft surface (1LSS)	Velocity (mean)		T1	<0.001	Higher in PG
one-legged stance on soft surface (1LSS)	Velocity (mean)		T3	0.038	Higher in PG
one-legged stance on soft surface (1LSS)	Velocity (mean) AP		T1	<0.001	Higher in PG
one-legged stance on soft surface (1LSS)	Velocity (mean) AP		T2	0.011	Higher in PG
one-legged stance on soft surface (1LSS)	Velocity (mean) AP		T3	0.006	Higher in PG
**(b)**					
**Stance**		**Occurence**			
one-legged stance on soft surface (1LSS)		8/21 (38% of all 1LSS tests)			
two-legged stance on soft surface (2LSS)		6/21 (29% of all 2LFS tests)			
two-legged stance on firm surface (2LFS)		4/21 (19% of all 2LSS tests)			
one-legged stance on firm surface (1LFS)		3/21 (14% of all 1LFS tests)			
tandem stance on soft surface (TanSS)		3/21 (14% of all TanSS tests)			

## 4. Discussion

This study presents the first investigation of the application of an instrumented BESS by combining a clinical balance assessment with posturographic measurements on behalf of a force plate in children and adolescents with acute mTBI sustained during regular everyday activities and starting as young as six years of age. It is also the first study to examine the trajectory of postural performance across three distinct timepoints early post injury in this age group. Furthermore, the study offers a comprehensive analysis of various posturographic outcome measures, incorporating comparisons with a control group.

Previous research has primarily focused on the reliability, validity, and feasibility of an instrumented BESS or was limited to singular outcome parameters such as VM when reporting instrumented data [30,50,51]. Although an instrumented BESS has been used to evaluate postural performance in pediatric and adult neurology populations, the existing literature exploring instrumented posturography is highly heterogeneous, characterized by varied assessment protocols and the use of different posturographic sensors [30,50,51,52,53]. This lack of uniformity hinders comparisons between cohorts and confines the clinical application of instrumented posturography in point-of-care settings. Thus, the importance of establishing standardized testing procedures to compile a comparable set of outcome parameters for future investigations cannot be overstated. This study contributes new clinical data for both patients and controls using an instrumented BESS, enriching the scientific discourse in the field of mTBI-related research.

### 4.1. BESS

The BESS total scores observed in both cohorts align with previous findings in this age group, with subscores increasing in accordance with the complexity of the testing positions [30,54,55]. Given the study set up with three assessments during the trajectory after mTBI, we hypothesized a decline in BESS errors over time in the PG and lower balance performance in the PG than in the CG, in particular at the early study visits. Interestingly, a significant reduction in errors was observed under only one condition—the two-legged stance on a soft surface. Additionally, this position—the two-legged stance on a soft surface—was the only one to reveal a significant in-between group difference, but with the PG scoring better than the CG scoring at the second and third BESS assessment during the study period.

So far, the trajectory of BESS performance following head trauma has only been studied in college athletes. Interestingly, in this cohort, BESS performance yielded baseline results as early as three to five days after the incident [56,57]. Previous cross-sectional investigations within the pediatric population reported significant differences in BESS testing between mTBI patients and controls. A 2014 study by Quatman-Yeates et al. evaluated BESS performance in 20 mTBI patients (13.24 ± 1.27 years of age) 7.45 ± 3.22 days post injury and 20 age- and sex-matched controls, reporting significantly lower BESS performance in the mTBI cohort in all BESS positions, except in the two-legged stances, and the overall BESS score [58]. Similarly, a 2015 study by Sambasivan et al. corroborated these findings, demonstrating significantly reduced total BESS scores in 26 children (13.32 ± 2.20 years of age) 42.88 ± 25.36 days after mTBI compared to 22 controls (13.59 ± 2.56 years of age) [59]. Yet these findings could not be substantiated in our cohort and study design, although we tested as early as in the first 72 h, 14 days, and 3 months after mTBI.

The following reasons may play a role here. First, the initial BESS scores of the PG align well with the scorings of the CG and with the normative data reported for healthy children and adolescents [55]. Thus, the patients most likely exhibited age-adequate postural control already at T1, leaving limited room for improvement. In this study, the first testing took place on average 30.5 h after the incident; maybe earlier testing, conducted directly during the initial presentation to the ED, would have demonstrated different scorings. Second, the findings of the current study may point to a limited discriminatory ability of the BESS as a clinical, examiner-rated assessment. Third, regarding the improvement under the two-legged soft surface condition, on the one hand, learning effects may play a role, as the PG underwent three testing sessions, whereas the CG was tested only once in a cross-sectional setup [40,60]. Thus, learning effects may compete with the natural improvement of symptoms throughout the recovery trajectory in the PG. To mitigate such possible learning effects, it has been recommended to conduct three trials within a single testing session and to average the results prior to analysis [60]. Yet in the pediatric population after mTBI, a carefully chosen testing set up with no risk of overexertion and taking reduced physical and cognitive capability into account has to be emphasized [30,54]. Therefore, we did not perform multiple testing during the same study visit. On the other hand, if learning effects would have confounded the BESS scores, this would unlikely have only struck through under one single test condition and would most probably have also affected performance under the more demanding ones. Fourth, while the BESS is a validated and widely used tool for assessing postural performance post-head injury, variability in BESS scores among younger cohorts has been reported, likely due to ongoing maturation of postural control during childhood [31,61,62,63]. The relatively young age of both cohorts in this study, compared to those in previous research, may have pronounced this variability, potentially reducing the diagnostic precision of the BESS protocol too [31]. Fifth, the overall burden of symptoms as reported within the PCSI shortly after the trauma may have been too low to cause apparent balance deficits, even if the distinct decline in the number and characteristics of symptoms reflected clinical recovery. As not any of the previous studies incorporated a quantitative assessment of the burden of symptoms, we cannot compare our cohort to theirs in this regard.

### 4.2. Instrumented BESS

To quantify balance performance, this study explored EA as a measure of the overall sway magnitude; PL, PL AP, and PL ML as indicators of sway trajectory; and VM, VM AP, and VM ML as indicators of body sway responsiveness. Similar to the BESS results, body swaying increased with the difficulty of testing positions in both groups, with the two-legged firm surface condition demonstrating the least and the one-legged soft surface condition the highest center of pressure displacements.

Although a decrease in postural swaying in the PG was anticipated in posturographic testing across the three-month follow-up timespan, the analysis revealed no consistent trends, with isolated significant differences lacking a clear pattern, which was likely attributed to high baseline functionality as observed during BESS testing and the probably relatively low burden of symptoms, as discussed above.

Comparisons of the posturographic measures of the PG at all three timepoints and the CG did not reveal consistent distinctions across all of the testing conditions. Despite this, several significant between-group differences emerged that may potentially highlight specific aspects of postural instability after mTBI. The conditions that most frequently demonstrated significant differences between the PG and the CG were the one-legged soft surface and two-legged soft surface, with 38% and 29% of the analyzed comparisons, respectively. These two conditions impose a high postural challenge due to the alteration of the proprioceptive information caused by the soft foam pad. Thus, such demanding conditions potentially demask postural instability in situations with limited capability of physiological integration of sensory, proprioceptive, and vestibular information, as after mTBI. This finding aligns with previous research that consistently identified the one-legged soft surface condition as a sensitive indicator of postural instability in clinical and instrumented BESS testing [30,40,58]. Less complex posturographic tasks may lack the power to sufficiently stress the mechanisms involved in postural control to detect alterations, also reflected in low outcomes in BESS and instrumented BESS testing in those positions, as shown in our data. Although these subtle alterations may go unnoticed in easier stances, they can still contribute to a functional impairment in daily activities, particularly in situations requiring dual tasking and shared attention. Such real-life scenarios, which often exacerbate postural instability, are not replicated in the artificial and very controlled environment of scientific research. All of the described circumstances highlight the importance of incorporating demanding postural tasks into balance assessments to enhance the detection of potentially clinically relevant impairments [19,40,64]. Further, future research may introduce dual task paradigms to balance assessments, evaluating not only static but also dynamic balance performance and gait, or introduce inertial sensors to track postural performance in the real-life settings of children and adolescents.

Although all reported measures in this study were derived from the displacement of the CoP, their diagnostic utility varies significantly. While PL and VM measures demonstrated some significant between-group differences in the presented study, EA did not reveal significant differences under any testing condition. This discrepancy likely arises from the fundamental differences in how the outcome measures are calculated: by mathematical definition, increased swaying will consistently result in a higher PL, making it a sensitive indicator of all postural shifts. By contrast, EA reflects swaying within the 90% ellipse, allowing fluctuations within this boundary without an increase unless swaying exceeds the perimeter. As a result, even frequent substantial body excursions within the ellipse may not alter EA, limiting its sensitivity to detect the entirety of postural responses. While extreme sway events detected by EA may often be visible during observational assessments like the BESS, subtle but frequent swaying captured by PL may be less apparent and thus benefit more from instrumented measurement.

Previous studies have reported significant alterations in postural control among mTBI patients compared to healthy controls as assessed using force plate sensors. For example, Chen et al. (2017) evaluated 244 adults 3.94 ± 1.4 days after mTBI and 105 healthy controls using the “postural stability test and modified clinical test of sensory integration in balance” and reported significant deficits in postural stability and sensory integration in the mTBI group [65]. Similarly, Geurts et al. (1996) observed reduced postural capabilities in adults with mTBI during quiet standing on a force plate even after a longer period after trauma (28.6 ± 25.7 months) [66]. Studies in pediatric populations, such as those by Rhine et al. (2016) and Gagnon et al. (2004), also identified significant alterations in postural control immediately after pediatric mTBI using various tools, including force plates and Wii Balance Boards [67,68]. King et al. (2014) utilized inertial sensors for an instrumented BESS in a pediatric cohort and found significant postural sway differences between mTBI patients (16.3 ± 2 years) 5 ± 3.3 months post trauma and controls (16.7 ± 2 years), with mTBI patients exhibiting significant larger sway areas [69]. Similarly, Abdul Rahman et al. (2019) reported significant postural impairments in children with TBI (11.63 ± 1.89 years, mean GCS of 9) using inertial posturography even 2.43 ± 1.48 years after trauma [52]. It is important to highlight that the studies by King et al. and Rahman et al., while examining a comparable age group, utilized different methods to assess postural control (inertial sensors versus static ground reaction force plates). This discrepancy complicates direct comparisons of the findings and emphasizes the need for standardized testing procedures. Further research exists that replicates measurable differences in cohorts affected by TBI of all severities against healthy controls with a wide array of different methodologies across different populations [70,71]. All of these findings highlight the pervasive impact of (m)TBI on postural control across diverse populations and methodologies. The current study replicates findings from prior research demonstrating some significant differences between mTBI patients and controls but not across all testing positions and all CoP outcome measures. There is evidence that postural performance improves with age [61,72]. Thus, the inconsistency may stem from the high variability in postural performance among younger children due to ongoing maturation processes [61,62,63,72,73]. Both cohorts in this study exhibited greater swaying in the medio–lateral direction compared to the anterior–posterior direction, a trend consistent with findings that younger children with less developed postural integration are more likely prone to higher mediolateral swaying [72]. Notably, a previous study conducted by Cochrane et al. (2021) observed also no consistent significant balance differences between 33 pediatric mTBI patients (13.1 ± 2.4 years) and 30 healthy controls (12.8 ± 3.0 years) using static posturography, attributing this to age-related variability and protocol inefficacies. While our results do not align with theirs in their entirety, they do provide additional evidence that postural maturation, as reflected by medio–lateral sway patterns, contributes to high variability and thus may lead to inconsistent differences [53]. Although a decrease in postural swaying in the PG was anticipated in posturographic testing across the three-month follow-up timespan, the analysis revealed no consistent trends, with isolated significant differences lacking a clear pattern, likely attributed to high baseline functionality and the low symptom burden of patients. Additionally, since patients lacked pre-trauma baseline data, the extent of mTBI effects on clinical balance performance cannot be accurately determined. Without a pre-mTBI reference, it remains challenging to determine whether the observed differences in postural performance stem from trauma-related deficits or natural inter-individual variability. In pediatric populations, this challenge is further pronounced by the ongoing maturation of the neuromuscular and sensory systems. To improve the clinical utility of postural assessments such as an instrumented BESS in pediatric TBI, future research should focus on establishing age-specific normative percentiles for posturographic measures. These normative data would provide a more accurate framework for interpreting individual results. In contexts where pre-injury data collection is feasible—such as among pediatric athletes or individuals undergoing routine physical evaluations—conducting baseline posturographic assessments could offer valuable reference points for post-injury comparisons, thereby improving diagnostic and monitoring precision. Future studies would benefit from incorporating sociodemographic factors, such as regular physical activity, including involvement in organized sports activities, to better contextualize these findings.

Integrating serial posturographic testing into post-mTBI management may enable clinicians to monitor functional recovery over time, thereby informing decisions regarding the safe continuation of physical activities and potentially supporting a more conservative return-to-play approach where necessary. Furthermore, given the variability in postural maturation among pediatric patients, rehabilitation strategies should incorporate age-specific balance training interventions that enhance sensory integration, proprioception, and vestibular function [74]. Future research may investigate whether early, targeted balance interventions, such as dual task balance exercises or vestibular therapy, can reduce prolonged postural instability and facilitate a safer reintegration into daily activities and sports participation. By refining return-to-play protocols with objective postural metrics, clinicians can ensure that children recovering from mTBI do not resume high-risk activities prematurely, thereby minimizing the risk of reinjury and prolonged recovery.

## 5. Strengths and Limitations

This study makes a significant contribution to the growing body of literature on pediatric mTBI by addressing a critical gap in the assessment of a key post-mTBI symptom. While existing research has disproportionately focused on more severe forms of TBI in adults, this study highlights the need for objective, instrumented methods to evaluate balance impairments in pediatric mTBI—an aspect often overlooked in neurotrauma management. Additionally, it helps to bridge the gap in meeting the unmet needs of pediatric mTBI patients in major trauma centers, as identified by Dasic et al. (2022) [75]. By emphasizing the importance of standardized postural assessments, this study provides a foundation for future research aimed at enhancing diagnostic accuracy and improving clinical management strategies for pediatric mTBI. Taking all findings together, this study underscores the necessity of contextualizing the results from comparable studies and our own research within the broader framework of the highly dynamic neuro-developmental processes during childhood. The variability in performance in neurological assessments is particularly high in this age group and affords a thorough evaluation by experienced clinicians. This circumstance, together with limited ability to articulate symptoms and significant inter-individual differences in behavior and response to testing, reinforces the challenges in research and everyday practice in the pediatric cohort. In light of these encounters, our study sought to objectify the mTBI-related clinical pattern on behalf of the use of the PCSI and to enhance the reliability of the outcomes by ensuring that all evaluations were conducted under supervision by a child neurologist and a specifically trained research assistant.

Overall, the scientific landscape regarding the instrumentation of balance assessments remains heterogeneous, and selecting comprehensive balance protocols that reflect current scientific discussions remains challenging. Establishing consistent protocols for balance instrumentation is paramount for future studies, particularly in younger cohorts like the one examined in this study. It is unrealistic to expect young children to perform uniformly across various testing procedures [53]. In addition, the point-of-care setting usually does not allow for too extensive testing. Therefore, the primary objective should be to devise short and tailored protocols incorporating the most promising conditions and catering to the developmental stage of patients of all ages, ensuring more reliable results in future investigations. When interpreting the results of instrumented posturography, it is essential to account for various confounding factors that may obscure the effects of mTBI on postural performance. Motor learning effects represent a significant consideration, particularly in longitudinal study designs such as the one employed in this investigation. Repeated assessments may lead to performance improvements independent of neurological recovery, potentially masking postural deficits [43]. In this study, the PG underwent three testing sessions, while the CG was assessed only once, raising the possibility of practice effects influencing the outcomes. Although averaging multiple trials within a single session has been proposed as a strategy to mitigate learning effects, the pediatric population requires a testing protocol that minimizes cognitive and physical strain, as outlined above [60]. Regarding motor learning, previous research suggests that significant motor learning effects typically require repeated practice with a high number of trials, often exceeding 100 repetitions within a short time frame, such as one week. In our study, the time intervals between assessments ranged from two weeks to over two months, making it unlikely that motor learning had a meaningful impact on postural performance. However, while we cannot entirely rule out motor learning effects, we expect them to be minimal. This perspective is supported by the existing literature, including van Abswoude et al. (2021), who reviewed implicit motor learning in primary school children [76].

Additionally, fatigue may adversely affect postural stability, particularly under more challenging test conditions [77]. Prolonged exertion can lead to compensatory adjustments that artificially elevate sway parameters, as previously described in various adult populations [78,79]. Attentional drift, which is particularly pronounced in younger children, further complicates the interpretation of postural control metrics. Fluctuations in focus during static balance assessments may introduce variability in postural sway measurements, especially under conditions requiring sustained concentration [30,53,68].

The test protocol of the BESS has a total duration of approximately four minutes. Given this short duration, attentional drift in children aged 6 to 17 years is expected to be minimal. Yet given that balance performance is modulated by multisensory integration and attentional control, even minor lapses in engagement during testing could contribute to the observed variability in postural control outcomes. These factors highlight the importance of implementing standardized testing protocols that address potential confounders such as learning effects, fatigue, and attentional influences. The sample size and single-center design of our study furthermore limit the generalizability of this study’s findings. It is essential to note that the primary objective of this study was not to validate the instrumented BESS against the BESS, but the BESS protocol was rather utilized as a standardized approach for posturographic testing. Still, we found no expected differences between the PG and CG in BESS testing, while the instrumented BESS found a set of significant differences, pointing to a higher potential for discrimination. This aspect should be further investigated in future studies.

## 6. Conclusions

This study highlights the potential of an instrumented BESS to reveal some subtle differences in postural control after mTBI and during the following trajectory compared to singular clinical BESS testing. Instrumented point-of-care assessments should focus on more demanding balance conditions that in particular affect the perception and processing of sensory-proprioceptive information. Further, testing protocols should account for age-dependent factors and challenges to achieve the best possible diagnostic precision. Future studies are needed to assess balance impairments as early as during the initial presentation to the to explore postural performance in larger samples and to explore postural performance in larger samples of patients with different levels of acute symptom burden. In addition, future research should prioritize the development of more dynamic balance assessments that mimic real-life postural requirements in a better way.

## Figures and Tables

**Table 2:** BESS subscores.

	Patient Group T1		Patient Group T2		Patient Group T3		Control	
	BESS Score		BESS Score		BESS Score		BESS Score	
	Mean (sd)	Median (iqr)	Mean (sd)	Median (iqr)	Mean (sd)	Median (iqr)	Mean (sd)	Median (iqr)
two-legged stance on firm surface	0.35 (1.77)	0 (0)	0.32 (0.18)	0 (0)	0.07 (0.25)	0 (0)	0	0
tandem stance on firm surface	2.55 (2.61)	2 (4)	1.77 (2.32)	1 (2.5)	1.63 (1.92)	1 (3)	1.61 (1.64)	1 (1)
one-legged stance on firm surface	4.94 (3.12)	4 (5)	4.19 (3.20)	4 (3.5)	4.23 (2.92)	4 (4)	5.10 (2.88)	5 (4)
total of positions on firm surface	7.84 (6.22)	6 (7.5)	6.23 (4.93)	5 (6.5)	5.93 (4.31)	6 (6.75)	6.71 (3.84)	6 (6.5)
two-legged stance on soft surface	1.35 (2.55)	0 (1.5)	0.55 (1.81)	0 (0)	0.20 (0.54)	0 (0)	0.94 (1.05)	1 (1)
tandem stance on soft surface	5.52 (3.20)	6 (5)	4.71 (3.35)	4 (5.5)	4.9 (3.14)	5 (5.75)	5.03 (2.76)	5 (4.5)
one-legged stance on soft surface	7.84 (2.53)	9 (3.5)	8.19 (1.89)	8 (3)	8.03 (2.11)	9 (3.75)	8.61 (1.50)	9 (2)
total of positions on soft surface	14.71 (6.45)	15 (8.5)	13.45 (5.45)	12 (8)	13.13 (4.87)	13 (8)	14.58 (3.53)	15 (5)
BESS total	22.55 (11.88)	20 (14)	19.45 (9.84)	18 (11.5)	19.10 (8.45)	19 (14)	21.29 (5.78)	22 (9)

**Table 3:** Results of the instrumented BESS.

Patient Group T1								
		Path Length	Path Length AP	Path Length ML	Velocity (Mean)	Velocity (Mean) AP	Velocity (Mean) ML	Ellipse Area
two-legged stance on firm surface (2LFS)	mean (sd)	585.24 (454.92)	358.470 (330.63)	370.66 (254.984)	3.53 (4.07)	31.43 (72.47)	18.50 (12.74)	8.68 (17.91)
	median (iqr)	445.77 (404.31)	256.791 (307.20)	284.413 (211.40)	2.229 (2.23)	13.517 (16.90)	14.221 (9.90)	4.432 (5.40)
two-legged stance on soft surface (2LSS)	mean (sd)	1172.63 (333.63)	694.39 (274.79)	767.10 (210.36)	7.02 (6.67)	57.56 (121.39)	34.29 (10.51)	19.77 (9.41)
	median (iqr)	1211.69 (541.01)	754.11 (314.19)	819.30 (349.10)	6.25 (2.75)	39.17 (16.91)	40.83 (17.46)	19.58 (13.41)
tandem stance on firm surface (TanFS)	mean (sd)	1473.10 (692.61)	1024.16 (623.76)	768.81 (277.29)	8.48 (5.38)	63.24 (48.90)	38.98 (13.98)	24.19 (20.08)
	median (iqr)	1313.03 (1042.81)	758.13 (904.05)	712.52 (445.88)	6.93 (6.02)	40.54 (50.88)	36.78 (23.00)	13.95 (30.78)
tandem stance on soft surface (TanSS)	mean (sd)	2696.60 (1448.08)	1965.76 (1289.49)	1313.89 (554.017)	14.15 (7.99)	107.26 (68.99)	68.77 (30.27)	65.19 (50.77)
	median (iqr)	2423.47 (2701.33)	1593.47 (2371.94)	1320.59 (905.33)	12.12 (13.94)	83.92 (118.27)	68.91 (47.97)	48.79 (88.68)
one-legged stance on firm surface (1LFS)	mean (sd)	2156.49 (899.19)	1322.04 (717.64)	1289.81 (412.93)	12.26 (7.37)	81.33 (59.14)	64.41 (20.59)	51.04 (80.81)
	median (iqr)	1966.11 (1098.07)	1130.73 (832.70)	1210.88 (675.58)	10.25 (6.06)	66.79 (47.91)	60.54 (33.24)	29.00 (27.49)
one-legged stance on soft surface (1LSS)	mean (sd)	2983.03 (1040.22)	1720.07 (674.15)	1979.89 (773.77)	16.07 (8.56)	97.64 (56.12)	99.52 (38.84)	88.56 (60.21)
	median (iqr)	2772.93 (1682.33)	1712.89 (891.73)	1856.35 (1232.98)	13.86 (9.24)	88.18 (47.20)	92.82 (61.65)	71.42 (71.44)
**Patient Group T2**								
		Path Length	Path Length AP	Path Length ML	Velocity (Mean)	Velocity (Mean) AP	Velocity (Mean) ML	Ellipse Area
two-legged stance on firm surface (2LFS)	mean (sd)	560.10 (144.97)	369.27 (129.82)	392.77 (87.63)	2.81 (2.14)	21.84 (34.42)	20.48 (4.48)	4.98 (4.01)
	median (iqr)	538.73 (171.70)	266.72 (176.51)	293.00 (99.31)	2.80 (0.91)	13.80 (9.10)	20.65 (5.51)	3.59 (3.70)
two-legged stance on soft surface (2LSS)	mean (sd)	1225.63 (359.49)	698.53 (303.55)	803.71 (249.72)	8.37 (9.54)	41.17 (23.64)	39.79 (12.23)	18.93 (10.16)
	median (iqr)	1161.09 (359.49)	669.74 (340.80)	756.18 (240.33)	5.80 (3.18)	36.03 (21.36)	37.81 (11.41)	16.45 (12.16)
tandem stance on firm surface (TanFS)	mean (sd)	1514.31 (817.71)	957.99 (578.82)	821.75 (347.75)	8.35 (5.26)	105.98 (179.88)	55.29 (46.43)	29.79 (46.66)
	median (iqr)	1250.69 (983.95)	865.99 (688.54)	710.92 (437.60)	6.46 (5.07)	48.54 (42.08)	37.41 (27.11)	14.25 (25.15)
tandem stance on soft surface (TanSS)	mean (sd)	3108.03 (1904.68)	2196.73 (1587.93)	1931.35 (1240.89)	19.24 (14.22)	115.24 (75.48)	97.42 (64.14)	90.27 (96.74)
	median (iqr)	2450.17 (3253.16)	1789.49 (2577.87)	1720.03 (1732.04)	14.59 (20.26)	94.52 (121.85)	74.85 (100.65)	48.67 (126.92)
one-legged stance on firm surface (1LFS)	mean (sd)	2071.34 (672.79)	1278.04 (605.06)	1288.11 (379.48)	11.06 (5.23)	70.77 (38.39)	65.16 (19.11)	31.07 (19.44)
	median (iqr)	2095.31 (942.86)	1125.37 (638.42)	1330.03 (547.29)	10.79 (5.56)	62.23 (32.85)	70.64 (27.36)	30.37 (26.30)
one-legged stance on soft surface (1LSS)	mean (sd)	3542.29 (1218.53)	1758.75 (899.13)	2464.98 (901.55)	21.74 (18.68)	107.41 (60.19)	119.26 (41.19)	105.44 (74.91)
	median (iqr)	3428.14 (2035.64)	1754.09 (1264.22)	2288.84 (1626.45)	17.15 (10.44)	97.47 (50.95)	114.44 (78.44)	78.49 (117.04)
**Patient Group T3**								
		Path Length	Path Length AP	Path Length ML	Velocity (Mean)	Velocity (Mean) AP	Velocity (Mean) ML	Ellipse Area
two-legged stance on firm surface (2LFS)	mean (sd)	417.982 (149.11)	278.324 (110.60)	293.066 (125.91)	2.231 (0.896)	13.916 (5.53)	14.653 (6.31)	4.349 (3.41)
	median (iqr)	379.297 (251.30)	262.318 (178.30)	252.627 (177.50)	2.036 (1.30)	13.116 (8.90)	12.631 (8.90)	3.5 (4.40)
two-legged stance on soft surface (2LSS)	mean (sd)	1084.96 (272.68)	726.68 (195.10)	646.98 (164.17)	5.67 (1.89)	37.67 (12.94)	34.07 (11.49)	16.38 (7.91)
	median (iqr)	1044.03 (437.48)	740.98 (333.08)	631.03 (221.39)	5.37 (2.27)	37.08 (17.37)	32.69 (11.87)	13.74 (11.99)
tandem stance on firm surface (TanFS)	mean (sd)	1358.27 (673.48)	881.54 (446.61)	789.94 (301.55)	6.79 (3.37)	47.41 (28.53)	39.58 (15.04)	19.71 (23.22)
	median (iqr)	1127.51 (522.61)	721.76 (437.96)	736.79 (301.55)	5.64 (2.61)	36.74 (22.60)	36.84 (15.97)	11.63 (15.13)
tandem stance on soft surface (TanSS)	mean (sd)	2878.61 (1396.91)	1767.15 (888.69)	1969.34 (1012.14)	14.39 (6.98)	88.36 (44.43)	97.74 (51.18)	74.26 (54.67)
	median (iqr)	2453.79 (2566.55)	1546.51 (1562.59)	1666.08 (1789.11)	12.27 (12.83)	77.32 (78.13)	83.30 (93.16)	65.63 (77.23)
one-legged stance on firm surface (1LFS)	mean (sd)	2303.62 (1027.97)	1488.12 (755.07)	1405.69 (535.89)	11.52 (5.15)	74.46 (37.75)	69.54 (27.12)	47.77 (56.58)
	median (iqr)	1893.56 (1224.95)	1214.77 (1067.01)	1330.13 (561.75)	9.47 (6.12)	60.74 (53.35)	60.86 (28.75)	25.15 (39.72)
one-legged stance on soft surface (1LSS)	mean (sd)	3283.58 (996.91)	1945.62 (586.51)	2210.59 (701.09)	16.42 (4.98)	99.28 (29.33)	109.97 (35.03)	105.44 (80.29)
	median (iqr)	2999.80 (1542.25)	1803.36 (988.38)	2143.58 (1155.48)	14.99 (7.71)	98.17 (49.42)	104.36 (57.77)	70.12 (107.55)
**Control Group**								
		Path Length	Path Length AP	Path Length ML	Velocity (Mean)	Velocity (Mean) AP	Velocity (Mean) ML	Ellipse Area
two-legged stance on firm surface (2LFS)	mean (sd)	556.46 (134.63)	323. 69 (88.08)	381 0.26 (98.74)	2.78 (0.67)	16.18 (4.40)	19.06 (4.94)	6.06 (3.36)
	median (iqr)	581.17 (251.30)	313.23 (139.88)	380.62 (163.24)	2.91 (1.26)	15.67 (6.99)	19.03 (8.16)	5.50 (4.59)
two-legged stance on soft surface (2LSS)	mean (sd)	1080.47 (296.67)	593.33 (239.12)	632.89 (202.90)	5.11 (1.57)	43.55 (10.73)	37.97 (12.25)	12.66 (10.80)
	median (iqr)	1035.17 (391.67)	531.93 (331.45)	593.09 (344.04)	5.15 (2.65)	30.94 (16.56)	35.89 (11.79)	10.28 (8.87)
tandem stance on firm surface (TanFS)	mean (sd)	1337.37 (429.48)	789.86 (382.28)	759.92 (277.53)	5.35 (2.60)	43.07 (22.51)	31.75 (12.51)	15.13 (17.52)
	median (iqr)	1321.66 (745.39)	792.70 (530.65)	756.79 (461.72)	5.71 (4.49)	41.48 (32.61)	31.73 (22.11)	10.51 (17.76)
tandem stance on soft surface (TanSS)	mean (sd)	2307.21 (862.49)	1706.51 (901.74)	1380.19 (371.37)	13.49 (5.17)	95.63 (45.79)	67.46 (862.49)	61.78 (44.18)
	median (iqr)	2288.06 (1626.89)	1657.21 (1353.92)	1382.64 (583.49)	13.27 (8.66)	82.86 (43.32)	68.23 (29.23)	47.76 (65.03)
one-legged stance on firm surface (1LFS)	mean (sd)	2053.41 (699.30)	1273.48 (696.70)	1329.32 (855.91)	11.15 (3.63)	71.77 (35.73)	64.83 (23.61)	51.03 (145.45)
	median (iqr)	2080.46 (1142.11)	1526.87 (1108.26)	1317.49 (530.73)	10.22 (6.58)	60.34 (53.83)	62.35 (26.15)	43.65 (45.88)
one-legged stance on soft surface (1LSS)	mean (sd)	2379.67 (538.81)	1405.43 (566.14)	2054.28 (691.32)	15.45 (4.40)	93.17 (34.08)	104.21 (37.90)	107.61 (48.92)
	median (iqr)	2473.94 (571.32)	1400.26 (452.41)	2089.09 (878.07)	14.64 (4.92)	91.47 (22.86)	100.33 (46.61)	107.29 (65.42)

## Data Availability

The data that support the findings of this study are available upon request from Dr. med. Johanna Wagner (mail to: Johanna.Wagner@med.uni-muenchen.de). The data are not publicly available due to privacy or ethical restrictions.

## Supplementary Materials

The following supporting information can be downloaded at: https://www.mdpi.com/article/10.3390/jcm14051666/s1, Table S1: Within- and between group analysis: two-legged stance on firm surface, Table S2: Within- and between group analysis: two-legged stance on soft surface, Table S3: Within- and between group analysis: tandem stance on firm surface, Table S4: Within- and between group analysis: tandem stance on soft surface, Table S5: Within- and between group analysis: one-legged stance on firm surface, Table S6: Within- and between group analysis: one-legged stance on soft surface, Table S7: Within- and between group analysis: BESS, Table S8: Within-age group post hoc analysis for PCSI.

## Abbreviations

The following abbreviations are used in this manuscript:

BESS	Balance Error Scoring System
CISG	Concussion in Sport Group
CG	control group
CoP	center of pressure
EA	ellipse area
1LFS	firm surface, one-legged stance on the non-dominant foot
2LFS	firm surface, two-legged stance–feet hip-width apart
1LSS	soft surface, one-legged stance
2LSS	soft surface, two-legged stance
mTBI	mild traumatic brain injury
MV	mean velocity
MV AP	mean velocity in anterior–posterior direction
MV ML	mean velocity in medio–lateral direction
NATA	National Athletic Trainers’ Association
PCSI	Post-Concussion Symptom Inventory
PG	patient group
PL	path length
PL AP	path length in anterior–posterior direction
PL ML	path length in medio–lateral direction
TanFS	firm surface, tandem stance with non-dominant foot in back
TanSS	soft surface, tandem stance with non-dominant foot in back
TBI	traumatic brain injury
