# Supplementary material for: Balance Performance After Mild Traumatic Brain Injury in Children and Adolescents: Instrumented BESS in the Acute Situation and Over Time
Source: J Clin Med. 2025 Feb 28;14(5):1666. doi: 10.3390/jcm14051666 (PMC11901075; doi:10.3390/jcm14051666)
Supplement: Supplementary file 1 [file jcm-14-01666-s001.zip › jcm-3460783-supplementary.pdf]

Supplemental Table S1: Within- and between group analysis: two-legged stance on firm surface

Variable	Within-Group F-statistic	Within-Group p-value	T1 vs Control T-test p-value	T1 vs Control Mann-Whitney U p-value	T2 vs Control T-test p-value	T2 vs Control Mann-Whitney U p-value	T3 vs Control T-test p-value	T3 vs Control Mann-Whitney U p-value
Path Length	2.590	0.810	0.742	0.135	0.007*	**0.011***	<0.001*	**<0.001***
Path Length AP	1.525	0.224	0.581	0.168	0.660	0.059	0.880	0.600
Path Length ML	2.019	0.139	0.833	0.061	< 0.001*	**< 0.001***	< 0.001*	**< 0.001***
Velocity (mean)	1.683	0.192	0.328	0.173	0.956	0.950	0.060	0.060
Velocity (mean) AP	1.054	0.353	0.253	0.469	0.387	0.255	0.115	0.880
Velocity (mean) ML	1.946	0.149	0.821	0.071	0.064	0.065	0.081	0.062
Ellipse Area	1.385	0.256	0.438	0.309	0.262	0.540	0.560	0.511

Supplemental Table S2: Within- and between group analysis: two-legged stance on soft surface

Variable	Within-Group F-statistic	Within-Group p-value	T1 vs Control T-test p-value	T1 vs Control Mann-Whitney U p-value	T2 vs Control T-test p-value	T2 vs Control Mann-Whitney U p-value	T3 vs Control T-test p-value	T3 vs Control Mann-Whitney U p-value
Path Length	1.409	0.250	0.130	0.400	0.070	**0.045***	< 0.001*	**0.001***
Path Length AP	1.320	0.876	0.710	0.192	0.122	0.141	0.910	0.210
Path Length ML	4.421	0.015*	**0.003***	0.014*	0.034*	**0.016***	< 0.001*	**< 0.001***
Velocity (mean)	1.181	0.312	0.938	0.361	0.482	0.630	< 0.001*	**< 0.001***
Velocity (mean) AP	.648	0.526	0.498	0.112	0.779	0.123	0.129	0.800
Velocity (mean) ML	1.994	0.052	0.061	0.091	0.131	0.070	0.051	0.059
Ellipse Area	1.086	0.342	0.317	0.391	0.215	0.129	0.271	0.121

Supplemental Table S3: Within- and between group analysis: tandem stance on firm surface

Variable	Within-Group F-statistic	Within-Group p-value	T1 vs Control T-test p-value	T1 vs Control Mann-Whitney U p-value	T2 vs Control T-test p-value	T2 vs Control Mann-Whitney U p-value	T3 vs Control T-test p-value	T3 vs Control Mann-Whitney U p-value
Path Length	0.355	0.702	0.464	0.922	0.378	0.728	0.995	0.662
Path Length AP	0.480	0.621	0.806	0.794	0.810	0.704	0.341	0.262
Path Length ML	0.207	0.814	0.229	0.235	0.654	0.476	0.374	0.363
Velocity (mean)	1.133	0.327	0.314	0.981	0.377	0.873	0.491	0.325
Velocity (mean) AP	2.325	0.104	0.311	0.994	0.115	0.590	0.407	0.222
Velocity (mean) ML	2.828	0.650	0.445	0.414	0.128	0.803	0.566	0.485
Ellipse Area	0.733	0.483	0.426	0.403	0.293	0.963	0.940	0.755

Supplemental Table S4: Within- and between group analysis: tandem stance on soft surface

Variable	Within-Group F-statistic	Within-Group p-value	T1 vs Control T-test p-value	T1 vs Control Mann-Whitney U p-value	T2 vs Control T-test p-value	T2 vs Control Mann-Whitney U p-value	T3 vs Control T-test p-value	T3 vs Control Mann-Whitney U p-value
Path Length	0.492	0.613	0.381	0.667	0.890	0.432	0.147	0.427
Path Length AP	0.846	0.433	0.845	0.828	0.400	0.872	0.577	0.572
Path Length ML	4.150	0.020*	0.612	0.484	**0.030***	0.357	**0.005***	0.104
Velocity (mean)	2.353	0.101	0.715	0.980	**0.047***	0.331	0.587	0.886
Velocity (mean) AP	1.369	0.260	0.465	0.855	0.253	0.636	0.564	0.572
Velocity (mean) ML	3.281	0.070	0.846	0.089	0.069	0.083	0.071	0.110
Ellipse Area	0.931	0.398	0.797	0.936	0.172	0.640	0.367	0.406

Supplemental Table S5: Within- and between group analysis: one-legged stance on firm surface

Variable	Within-Group F-statistic	Within-Group p-value	T1 vs Control T-test p-value	T1 vs Control Mann-Whitney U p-value	T2 vs Control T-test p-value	T2 vs Control Mann-Whitney U p-value	T3 vs Control T-test p-value	T3 vs Control Mann-Whitney U p-value
Path Length	0.518	0.597	0.406	0.246	181	0.273	0.844	0.399
Path Length AP	1.112	0.334	0.019*	**0.009***	0.003*	**< 0.001***	0.131	0.660
Path Length ML	0.652	0.524	0.750	0.760	0.690	0.740	0.258	0.225
Velocity (mean)	0.289	0.749	0.804	0.132	0.212	0.740	0.359	0.131
Velocity (mean) AP	0.390	0.678	0.520	0.059	0.071	**0.028***	0.135	0.630
Velocity (mean) ML	0.433	0.650	0.950	0.106	0.108	0.149	0.447	0.235
Ellipse Area	0.987	0.377	0.491	0.740	0.173	0.310	0.412	0.185

Supplemental Table S6: Within- and between group analysis: one-legged stance on soft surface

Variable	Within-Group F-statistic	Within-Group p-value	T1 vs Control T-test p-value	T1 vs Control Mann-Whitney U p-value	T2 vs Control T-test p-value	T2 vs Control Mann-Whitney U p-value	T3 vs Control T-test p-value	T3 vs Control Mann-Whitney U p-value
Path Length	1.946	0.149	0.103	132	0.548	0.980	0.687	0.516
Path Length AP	0.775	0.464	< 0.001*	**< 0.001***	0.003*	**< 0.001***	0.007*	**< 0.001***
Path Length ML	2.730	0.710	0.710	0.100	0.691	0.580	0.890	0.940
Velocity (mean)	1.949	0.149	0.720	**< 0.001***	0.531	0.359	0.028*	**0.038***
Velocity (mean) AP	0.359	0.700	0.025*	**< 0.001***	0.170	**0.011***	0.002*	**0.006***
Velocity (mean) ML	1.944	0.150	0.140	0.210	0.167	0.184	0.180	0.220
Ellipse Area	0.538	0.586	0.800	0.100	0.513	0.212	0.526	0.200

Supplemental Table S7: Within- and between group analysis: BESS

Condition	Within-Group F-statistic	ANOVA p-value	T1 vs Control Test Type	T1 vs Control p-value	T2 vs Control Test Type	T2 vs Control p-value	T3 vs Control Test Type	T3 vs Control p-value
two-legged stance on firm surface	0.881	0.418	Mann-Whitney U-Test	0.161	Mann-Whitney U-Test	0.333	Mann-Whitney U-Test	0.161
two-legged stance on soft surface	3.171	.047*	Mann-Whitney U-Test	0.552	Mann-Whitney U-Test	< 0.001*	Mann-Whitney U-Test	< 0.001*
tandem stance on firm surface	1.423	.246	Mann-Whitney U-Test	0.296	Mann-Whitney U-Test	0.624	Mann-Whitney U-Test	0.661
tandem stance on soft surface	0.59	0.557	T-Test	0.533	Mann-Whitney U-Test	0.656	T-Test	0.709
one-legged stance on firm surface	0.632	0.534	Mann-Whitney U-Test	0.702	Mann-Whitney U-Test	0.173	T-Test	0.202
one-legged stance on soft surface	0.199	0.820	Mann-Whitney U-Test	0.449	Mann-Whitney U-Test	0.503	Mann-Whitney U-Test	0.369
BESS Total	1.115	0.332	T-Test	0.604	Mann-Whitney U-Test	0.109	T-Test	0.210

Supplemental Table S8: Within-age group post-hoc analysis for PCSI

Age Group	T1 vs T2		T1 vs T3		T2 vs T3	
	p- value	median difference	p- value	median difference	p- value	median difference
**8 - 12 years**	0.001	10	0.001	10	2.47	0
**12 - 18 years**	0.552	13.5	0.312	15.5	2.16	2

Note: The 12–18 years age group presents a more variable recovery. While significant differences across timepoints were detected, pairwise post-hoc comparisons did not reveal statistically significant symptom reductions. This lack of significant change, despite observed trends in symptom resolution by T3, may highlight greater heterogeneity in post-concussion recovery trajectories during adolescence, also reflected by the high standard deviations observed in our data in this age-group.
